# Complete Obturation—Cold Lateral Condensation vs. Thermoplastic Techniques: A Systematic Review of Micro-CT Studies

**DOI:** 10.3390/ma14144013

**Published:** 2021-07-18

**Authors:** Shilpa Bhandi, Mohammed Mashyakhy, Abdulaziz S. Abumelha, Mazen F. Alkahtany, Mohamed Jamal, Hitesh Chohan, A. Thirumal Raj, Luca Testarelli, Rodolfo Reda, Shankargouda Patil

**Affiliations:** 1 Department of Restorative Dental Sciences, College of Dentistry, Jazan University, Jazan 45412, Saudi Arabia; shilpa.bhandi@gmail.com (S.B.); dr.mashyakhy@gmail.com (M.M.); drhiteshchohan@yahoo.co.in (H.C.); 2 Department of Restorative Dental Science, College of Dentistry, King Khalid University, Abha 61421, Saudi Arabia; aabumelha@kku.edu.sa; 3 Department of Restorative Dental Science, Division of Endodontics, College of Dentistry, King Saud University, Riyadh 11362, Saudi Arabia; malkahtany@ksu.edu.sa; 4 Department of Endodontics, Hamdan Bin Mohamed College of Dental Medicine, Mohammed Bin Rashid University of Medicine and Health Sciences, Dubai Health Care City, Dubai 505055, United Arab Emirates; mohamed.jamal@mbru.ac.ae; 5 Department of Oral Pathology and Microbiology, Sri Venkateswara Dental College and Hospital, Chennai 600130, India; thirumalraj666@gmail.com; 6 Department of Oral and Maxillo Facial Sciences, University of Rome La Sapienza, 00161 Rome, Italy; luca.testarelli@uniroma1.it (L.T.); rodolforeda17@gmail.com (R.R.); 7 Department of Maxillofacial Surgery and Diagnostic Sciences, Division of Oral Pathology, College of Dentistry, Jazan University, Jazan 45142, Saudi Arabia

**Keywords:** obturation, root canal filling, micro-CT, cold lateral condensation, thermoplasticized gutta-percha

## Abstract

To prevent re-infection and provide a hermetic seal of the root canal system, an endodontist must aim to produce a void-free obturation. This review aimed to compare the completeness of root canal obturation between the two most prevalent methods—cold lateral condensation and warm gutta-percha techniques—using micro-CT (PROSPERO reg no. 249815). Materials and Methods: A search of Scopus, Embase, PubMed (Medline via PubMed), and Web of Science databases was done without any time restriction according to the PRISMA protocol. Articles that compared both techniques and were published in English were included. Data was extracted and the risk of bias was assessed using an adapted tool based on previous studies. Results: A total of 141 studies were identified by the search. Following the screening and selection of articles, 9 studies were included for review. Data was extracted manually and tabulated. Most studies had a moderate risk of bias. None determined operator skill in both methods before comparison. The data extracted from the included studies suggests that both techniques produce voids in the obturation. The thermoplasticized gutta-percha techniques may result in fewer voids compared to cold lateral condensation. Conclusion: Considering the limitations of the included studies, it was concluded that neither technique could completely obturate the root canal. Thermoplasticized gutta-percha techniques showed better outcomes despite a possible learning bias in favor of cold lateral condensation. Establishing operator skills before comparison may help reduce this bias.

## 1. Introduction

Good obturation is a key requirement in successful endodontic treatment [1]. Cleaning and shaping of the canals affects proper debridement and removal of tissue remnants—an essential step in endodontic therapy. However, an incomplete filling can jeopardize the success of root canal treatment. Inadequate endodontic filling is linked to the development of periapical pathology and considered a failure of the endodontic treatment [2,3,4]. In a systematic review, Ng et al., found four factors that significantly improve the outcome of primary root canal treatment, and one of these was the presence of a root filling with no voids [5].

An inadequate root filling is a contributor among other factors [6,7,8,9,10]. It has been demonstrated that bacteria are a primary cause of endodontic treatment failure [9,10,11,12]. Lack of a hermetic seal in the root canal system creates a favourable environment for bacterial proliferation, especially for facultative anaerobes [9,13,14]. Root canals also have a complex anatomy with the occurrence of oval shaped canals in more than 90% teeth in some samples [15]. Therefore, even standard protocols for cleaning and shaping—be it using rotary, reciprocating or the self-adjusting files—can leave areas of the root canal untouched [16,17]. These areas are dependent on the action of irrigants to eliminate bacteria. Along with mechanical cleaning and shaping procedures, irrigants reduce the number of bacteria in the root canals but do not eliminate them [18,19,20,21,22].

A reduced microbial load in the absence of a subsequent apical and coronal seal can cause recurrence of infection [13]. Persistent bacteria can use tissue remnants from unprepared areas as nutrients, leading to bacterial proliferation that is sustained if tissue fluids move into the canal from the periapical region [23,24]. This occurs by one or a combination of these mechanisms: the inability of the immune system to reach these bacteria, a supply of nutrients from the periapical region, and new bacteria entering from the coronal orifice [25]. Proponents of the multiple visit endodontic treatment favor the use of intra-canal medicaments to overcome this problem. However, it is ineffective in eliminating bacteria [26,27]. A dense, complete obturation prevents contact between bacteria and their nutrient source, thus reducing the possibility of re-infection and re-treatment.

### Literature Review

Obturation is the filling and sealing of a prepared root canal with a root canal sealer and a core material. The core material occupies space while the sealer flows to areas of irregularities or those unaffected by mechanical preparation. An obturation must achieve a high level of adaptability to the prepared canal walls and the filling material must penetrate the dentinal tubules, if possible [28]. Sealers are essential to form an impervious barrier between the core material and the root canal walls [29]. The importance of sealers was realized in the early 20th century when obturations with gutta-percha alone frequently led to apical periodontitis [30]. They can flow into areas where the core filling materials do not reach and help obtain an adequate seal regardless of the technique used [31]. Sealers can penetrate dentinal tubules and have inhibitory effects on *E. fecalis* [32,33]. However, it is possible that sealer in the dentinal tubules offers no advantage in achieving a hermetic seal in the root canal [34].

Though the process of obturation involves placing a filling material, preparation of the root canal to receive the filling starts during biomechanical preparation. Schilder advised that cleaning and shaping should be carried out as per the root canal anatomy and obturation technique. He described a continuously tapered tunnel preparation for root canals that are to be obturated with gutta-percha. The tunnel tapers apically and must closely follow the shape of the original canal [35]. There are unprepared areas with modern cleaning and shaping systems, though to a varying degree with each [16,17,36]. They prevent complete obturation regardless of the technique by hindering adaptation of gutta-percha to the canal walls [37]. This led to a focus on cleaning and shaping methods as a single important factor in treating endodontic disease. However, such a view may be detrimental for long-term endodontic outcomes. Both canal preparation and filling need to be considered in tandem to provide the best outcome for endodontic therapy.

The classic obturation technique, also primarily taught in undergraduate courses in most dental schools, is cold lateral condensation [38,39,40]. This technique involves placing a single cone of gutta-percha (GP) with sealer in the prepared root canal and adding secondary GP cones that are compacted together with the use of a spreader. The cones stay together due to frictional grip and the presence of a sealer [41]. Although a time-consuming procedure, lateral condensation is preferred due to its low cost and controlled placement of GP in the canal [39,40]. The final mass is not homogenous and consists of numerous GP cones pressed together with the sealer filling most spaces in between [42]. The concept of heating GP to obtain a uniform tri-dimensional obturation was introduced by Schilder in the 1960s [1,43]. He aimed to provide a technique that produced a homogenous, stable, compatible material adapted to the varied and complex anatomy of the root canal system. This technique condenses heated GP in the canal to adapt it to the prepared root canal walls. The method uses little amount of sealer. Since the introduction of Schilder’s technique, other procedures that use heated gutta-percha cones evolved: the continuous wave obturation, injectable gutta-percha, and carrier-based techniques [44,45].

Studies that compared these two techniques in vitro, used sectioning, radiographs, weighted acrylic blocks and the double chamber model to evaluate the completeness, density and microleakage—all of which play a role in successful obturation [46,47,48,49]. These comparisons indicated that thermoplastic techniques were superior to cold lateral condensation. Another study, which compared the filled area in both techniques using microscopic analysis, found that the void area with thermoplastic techniques was less than the void area in lateral condensation [37]. However, sectioning teeth is invasive, can result in artefacts, and destroys the subject tooth. The imaging techniques used provide a two dimensional picture of a three dimensional problem.

The field of dental research is evolving with the introduction of digital technology. This has been used to provide better patient care with less invasive methods and less intensive use of resources. These technologies help create a better workflow for clinicians and have also helped in basic research such as with stem cells [50,51]. These technologies can also be used in other clinical aspects of dental research such as in orthodontics where a digital workflow can aid in accurately studying factors such as rate of tooth movement [52]. With advances in imaging technology and endodontic techniques, one can better assess the quality of fillings in the root canals. Researchers no longer need to rely on subjective symptoms or be limited by superimpositions in two-dimensional images. The use of digital imaging, cone beam computed tomography (CBCT), micro-computed tomography (Micro-CT), and Magnetic Resonance (MRI) provide detailed assessment and evaluation of the work. CBCT has been used extensively in all areas of dentistry for three-dimensional imaging. However, due to the high radiation exposure, guidelines are in place for its clinical use in endodontics [53,54]. It is also ineffective to assess voids in endodontic filling due to the presence of artefacts [32]. There are methods that reduce, but not eliminate, artefact production in CBCTs. This is done by using appropriate settings for scanning and a metal artifact reduction algorithm [55,56]. The expression of artefacts is variable among different CBCT machines [57]. MRI also suffers from similar problems in addition to high cost and long scan times [58,59].

Micro-computed tomography (micro-CT) can visualize root canals and determine the extent of filling materials at different levels without damage to the subject teeth. It is a reliable method of 3D imaging of the root canal anatomy [60,61]. This sophisticated technique is used to evaluate modern methods and experimental studies of root canal preparation. Its high resolution makes it particularly useful for modern techniques, which emphasize minimal preparation to preserve as much root dentin as possible [62]. Micro-CT was initially used to study root canal anatomy and later, root canal preparation [36,62,63]. It shows a high correlation with histologic examination of root canal fillings [64]. Comparisons between micro-CT and CBCT images of filled root canals show a higher volumetric distortion and artefacts with CBCTs [65,66]. Using nano-CT as a reference, comparison of different CBCT machines and micro-CT demonstrated a significant distortion with all CBCT machines in filled root canals [57]. Micro-CT is considered as the gold standard in studies evaluating quality of root canal filling, root canal morphology, evaluation of canal preparation, and irrigation [65]. Therefore, studies that used micro-CT were chosen for inclusion in the review.

The aim of this review was to examine the evidence on the three-dimensional completeness of root canal fillings produced by cold lateral condensation versus the warm gutta-percha techniques of obturation.

## 2. Materials and Methods

This systematic review was conducted following the Preferred Reporting Items for Systematic Review (PRISMA) guidelines [67]. The review was submitted for PROSPERO registration (249815).

### 2.1. Inclusion Criteria

The studies were included according to the PICOS format as follows:

− Participants (P)—teeth that had undergone root canal preparation followed by endodontic obturation.
− Intervention (I)—obturation done using heated gutta-percha techniques.
− Comparison (C)—obturation of the root canal using the cold lateral condensation (CLC) technique.
− Outcome (O)—assessment of root canal filling or voids on a micro-CT.
− Studies (S)—studies that compared two or more techniques, one of which was cold lateral condensation and another which was a heated gutta-percha technique.
− Those studies that objectively assessed and compared the obturation using micro-CT were included in this review.

### 2.2. Exclusion Criteria

Those that did not report the volume or volume fractions of the entire root canal were excluded. Studies that did not compare the two methods of obturation and were only descriptive towards one were not included due to the inability to compare techniques. Narrative reviews, case reports, opinion pieces, conference abstracts, and letters to the editor were excluded from the review. Articles in languages other than English were excluded.

### 2.3. Focus Question

This review aimed to answer the question: “Does gutta-percha used with the cold lateral condensation and warm condensation techniques objectively produce complete obturation of the root canal radiographically in three dimensions?”

### 2.4. Search Strategy

The search was conducted according to the PRISMA extension guidelines for reporting literature searches [68]. The PubMed, Scopus, Embase, and Web of Science databases were searched on 3 April 2021, without placing any time restrictions. The details of the search queries placed in the databases are given in Table 1.

The results obtained from these queries were exported to EndNote (Clarivate™, Philadelphia, PA, USA). Any duplicates were removed using the software. This was followed by an initial screening of titles and abstracts to identify relevant studies. This was done independently by two reviewers. After resolution of any ambiguity by discussion with a third reviewer, the studies identified during the initial search were independently evaluated by both reviewers with full-text reading. Any disagreements in the inclusion of studies were resolved by discussion with a third reviewer. Those satisfying the inclusion criteria were hand searched for additional studies. A hand search of prominent journals, based on data analysed by independent researchers, was also done to identify any additional studies [69]. These included the (1) Journal of Endodontics, (2) International Endodontic Journal, and (3) Oral Surgery, Oral Medicine, Oral Pathology, Oral Radiology, and Endodontology.

Data extracted from the studies included the type of study, sample size, the method of warm vertical compaction used, the use of irrigants for smear layer removal, use of sealers, measurements made with micro-CT, their differences, and the results. The data was extracted by two reviewers independently, discussed, and tabulated. The details were tabulated to allow comparison between the studies regarding the completeness of obturation (Table 2).

### 2.5. Risk of Bias

A customized criteria for assessment was devised based on previous systematic reviews on in vitro studies, but were adapted to include relevant factors that affect the success of root canal treatment [70,71]. The criteria used are listed in Table 3. Evaluation of the risk of bias was made by two reviewers individually. Any ambiguity in the results was resolved by discussion with a third reviewer.

## 3. Results

### 3.1. Identification of Studies

**Figure 1:**
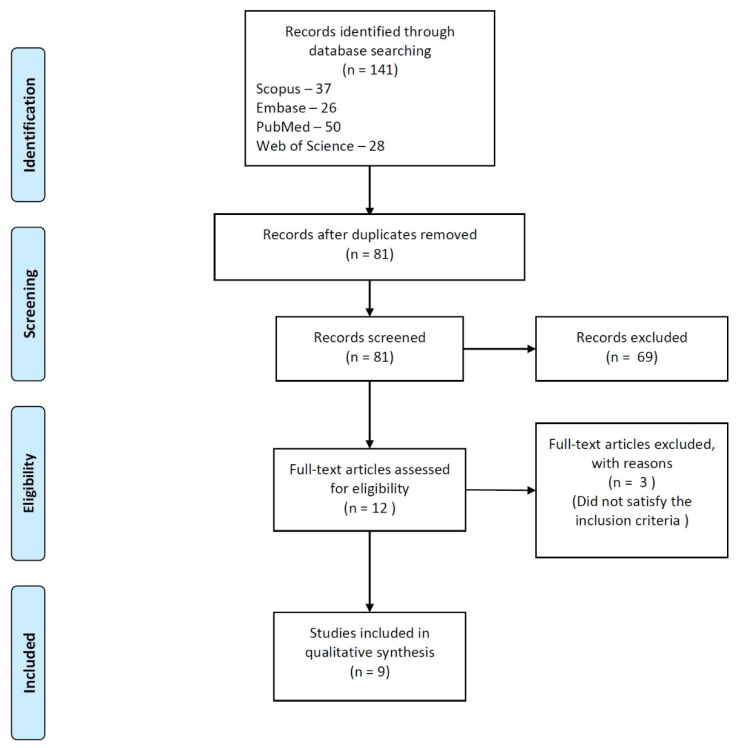
Summary of the workflow.

The workflow of the process used for study selection, according to the PRISMA guidelines, is presented in Figure 1.

An initial search of databases using the search terms revealed a total of 141 articles in the databases. After the removal of duplicates, 81 studies were screened using the titles and abstracts. The two reviewers involved in the screening were in almost perfect agreement (k = 0.90). The ambiguity was resolved by discussion with a third reviewer and a total of 12 articles were selected for full-text reading. Out of these articles, 3 did not satisfy the inclusion criteria [72,73,74]. Reviewer agreement was absolute (k = 1). The remaining articles were included in the review.

### 3.2. Assessment of Risk of Bias

The criteria used to assess the risk of bias were determined based on the methodological requirements to perform an experiment with control over possible factors in an in vitro environment. Factors such as the use of root canals of similar morphology, matching of groups, and the operator skill levels—all of which can influence obturation quality were considered. Standardization of the protocol for both groups was also taken into account. Of the included studies, only 1 had a low risk of bias. A maximum number of studies had a moderate risk of bias (6) and the remaining had a high risk (2).

### 3.3. Studies Examining the Completeness of Obturation

A total of nine studies were included for qualitative analysis [75,76,77,78,79,80,81,82,83]. These compared voids seen in obturation with thermoplastic techniques versus cold lateral condensation. The studies did not follow a uniform methodology for the assessment of the results but were able to objectively assess the outcome. Some used the volume of the voids present while others used the volume of the filling in the prepared canal. Measurements were made in the form of mean volumes of the filling or voids in similarly prepared canals or as volume percentages.

Neither technique produced a void-free obturation in the root canals. Of the nine studies examined, seven found significant differences between the two techniques, favouring the use of thermoplastic gutta-percha obturation techniques. However, two of these found significant differences only in the apical third [76,78]. Two studies found a greater number of voids with the thermoplastic technique but these differences were not significant [75,83]. A summary of the data extracted from the studies is given in Table 2.

## 4. Discussion

This study reviewed the completeness of obturation using heated gutta-percha in comparison to cold lateral condensation observed radiographically in three dimensions by micro-CT. We observed that heated gutta-percha techniques were a more favorable method of root canal obturation compared to the more widely taught cold lateral condensation but, further studies with better control over factors that can potentially influence the outcome are needed. The data extracted from the studies did not lend itself to quantitative analysis as the methods used for assessment varied among studies.

In vitro studies are done in a controlled environment and do not accurately resemble clinical settings. A number of variables such as patient’s oral and systemic health, level of co-operation; determinants related to teeth such as the case selection, type of pathology or tooth morphology and professional related conditions such as experience, stress or a new technology cannot be accounted for with in vitro studies. The assessment of bias in studies included in this review aimed to identify factors that could introduce bias in the research. This systematic review addressed studies that compared two different procedures of obturation. The use of extracted human teeth with complete root canal obturation were checked to create a condition that resembled clinical setting to an extent. The morphology of the canal can determine the presence of unprepared areas with different root canal preparation instruments. Thus, it was necessary that both groups were matched for canal morphology and cleaning and shaping procedures were similar for all teeth. To overcome bias related to operator skills, it was determined if the same operator performed both procedures and was adequately trained in them. For a significant comparison of the techniques, it is important that the sample size is calculated according to statistical methods and the groups are matched for characteristics such as canal length. The latter also helps eliminate selection bias among the groups. To avoid detection bias, it is necessary to blind the outcome assessor. Therefore, the nine criteria depicted in Table 3 were chosen to assess the risk of bias in the studies included in this review.

The included studies used micro-CT images to calculate voids with both obturation techniques. This non-invasive technique is inadequate to visualize filled root canals. It can be difficult to distinguish between materials, as the CT value of the same material may change at different locations and materials with close CT values can appear similar [84]. This makes detection problematic. To reduce these artifacts, attenuation filters can be used externally or optimal scanning parameters can be determined using different variables, i.e., imaging parameters and materials used. Mathematical filters such as a Gaussian filter in software can also be used to smoothen micro-CT images. Many studies in this review included a software that provides such mathematical filters [75,76,78,81,82]. One study used an external filter to control beam hardening artefacts [79]. Synchrotron facilities produce controlled and coherent X-ray beams depending on the spatial arrangement of magnetic fields. They help obtain micro-CT images with a combination of high resolution and different contrast modalities. The high resolution phase contrast X-ray imaging allows for observation in the sub-micrometer range and has been compared to histology in three dimensions [85]. In one study, these phase contrast enhanced (PCE) micro-CT reconstructions were compared to different methods for imaging endodontically treated teeth [86]. This study indicated that the PCE micro-CT was able to detect the GP, sealer, dentin and voids better than electron microscopy and laboratory micro-CT. The observers were unable to distinguish sealer from the GP in micro-CT images. It was seen that micro-CT underestimated areas compared to PCE micro-CT, which were attributed to dehydration during sample preparation. The micro-CT was especially unreliable to study the interface of dentin and root canal filling. The use of filters, external or mathematical, was not mentioned in this study. A disadvantage associated with the use of synchrotron facilities is the high cost and limited availability.

Studies used different methods to assess the completeness of root canal obturation, i.e., filling or voids, mean volumes, numbers, or size of voids—with many using multiple measurements. The void fractions or percentages were preferred as all studies did not distinguish between gutta-percha and sealer when considering the fill volume. When only fill volume was reported, the volume percentage was used instead of mean volumes to allow comparison regardless of the initial volume of root canal preparation. Removal of the smear layer is known to have a positive effect on the quality of obturation [87]. All studies in this review removed the smear layer before obturation except for one [81]. It is, therefore, not possible to state if the smear layer has any effect on the completeness of obturation with either technique.

An ideal obturation needs to be well adapted, void-free, provide an adequate seal for all canals connecting the pulp to the periodontium, and adapt to the prepared canal walls. [41] Neither technique can provide an obturation free of voids in the root canal according to the findings in this review. The quantitative measurement of voids differed among the techniques. The results of one of the studies differed from the rest—it found fewer voids in the cold lateral condensation group [75]. These differences were not significant. Two other studies that found no significant difference between the techniques did not measure the volume of voids directly but derived it based on cross-sections of the root [78,83]. One of these studies found significant difference in the apical third of the canal and fewer voids—internal and external—with the thermoplastic technique [78].

Studies examined the sealer volumes in the root canals [76,81] and found a greater proportion of sealer in the lateral condensation groups. Sealers undergo shrinkage after setting, which can contribute towards more voids observed in the lateral condensation groups. Another reason is the presence of spreader tracts in this group. These remnants do not fill up with sealer or GP. The warm gutta-percha techniques had lower volumes of voids between sealer and GP inside the filling as well as along the canal walls. This was superior to the lateral condensation and the single cone technique [78].

One study analysed the distribution of voids in a prepared canal, inside the filling (s-voids) and along the interface of the filling (i-voids) with the different obturation methods and cleaning and shaping techniques [79]. The lowest volume fraction and size of internal voids was observed with thermal techniques regardless of the system for cleaning and shaping—hand or rotary—but the voids were smaller in size with use of hand instrumentation. The number of voids at the interface was less with the thermal technique. The lowest volume of both types of voids was found with a combination of hand instrumentation and thermal obturation. However, in contrast to other studies [78,81], this study found more voids with the thermoplastic technique in the apical thirds of the root canal. Overall, there was a greater number of internal voids, but of a smaller size, with the thermoplastic technique. Voids at the dentin interface were higher in the lateral condensation group. The presence of voids at the interface may promote bacterial proliferation from untouched root areas and lead to failure of treatment. Another study compared the self-adjusting file system to rotary techniques [75] and found fewer voids with rotary instruments in both obturation techniques (though statistically insignificant). It is known that the technique of instrumentation affects the completeness of obturation [36]. The comparison of canal preparation and obturation as a whole with different combinations between techniques can be researched further. Establishing combinations of techniques better suited for use together will aid clinicians to make better decisions during treatment.

Despite the introduction of several methods of obturation, cold lateral condensation of gutta-percha and the vertical condensation of thermoplasticized GP are two widely prevalent techniques [40,88]. Lateral condensation has been considered as a gold standard for comparing newer obturation techniques to determine their efficacy and is recommended to obturate teeth with open apices [1,45,89]. The rationale behind the introduction of the heated gutta-percha (GP) technique was to allow the plasticized GP to adapt to the varied anatomy of the canal to provide a void-free filling [43]. Though theoretically possible, practical in vitro studies presented here do not confirm this. Studies that evaluated the two techniques and found significantly fewer voids in thermoplasticized GP also demonstrate that the filling of isthmuses and lateral canals occurs with the sealer [77,80].

The survival of an endodontically treated teeth depends on biological and mechanical factors. It cannot be denied that the dentist’s skill—diagnostic and practical technique—is essential for treatment success. Most practitioners are taught and clinically use the lateral condensation technique for obturation. Recent studies on the prevalence of obturation techniques show that this technique is more prevalent than warm vertical compaction in many clinical and teaching institutions [90,91,92,93]. Operator skills play an important role in the quality of obturation [5]. As a dentist becomes more experienced in a technique, he/she is more likely to provide an improved result. Only one study in this review [83] matched the operator’s skill level in both techniques. This could make the studies biased, most likely, in favour of the cold lateral condensation technique—which was demonstrated as inferior in most studies—and could also be a cause for the contradictory results in some studies. Future comparisons of the obturation techniques can help eliminate the bias by ensuring that the operator has equal experience in both techniques. Despite detailed protocol, results and skills between operators may vary [94]. Since this review addressed established techniques of obturation which were evaluated using a current imaging technique, a single operator with adequate training in both will allow better comparison of the techniques by not introducing variation in skill levels.

Previous in vitro studies comparing these two techniques found an increased incidence of apical extrusion of gutta-percha with the thermal techniques of obturation [46,89], others showed no difference [95] or a higher incidence with the lateral condensation techniques [88]. A meta-analysis of randomized controlled trials by Peng et al., showed a higher incidence of extrusion with the thermal techniques but the factors that could avoid this were operator-skill related—accurate determination of the working length, avoiding the destruction of the apical foramen, and controlling the insertion rate of warm GP [96]. None of the studies in this review reported the over extrusion of gutta-percha. This could be due to increased experience of dental professionals with the thermal techniques—resulting in better understanding compared to when it was newly introduced—or the development of modern, convenient tools for thermoplasticised GP obturation.

Recently, the idea of minimal preparation of root canals is becoming popular [45,62]. In one study, the preparation of the root canals with instruments of similar taper and differing apical enlargement found a higher amount of untouched dentin with the less invasive method (apical enlargement to 0.25 mm) [97]. These findings make it important that the subsequent steps in endodontic treatment are more reliable and predictable to ensure treatment success with minimally invasive techniques. Technological innovations have led to the development of materials with properties such as the ability to chemically bond with dentin. These might be adopted with minimally invasive techniques. In this review, two included studies compared filling materials that were not gutta-percha dependent. One study compared Ortho-MTA [76] and the other GuttaFlow [82] to conventional obturation techniques. Both produced significantly fewer voids than the cold lateral condensation group but the difference between these two methods and the thermoplasticized gutta-percha techniques was not significant. Ortho-MTA was able to flow into the canal isthmus, which was accomplished by the sealer in thermal compaction technique and not at all in the cold lateral condensation technique. Neither of the two—OrthoMTA or GuttaFlow—was able to produce void-free obturation.

Gutta-percha might not have the ideal properties of an obturating material according to Grossman’s or Sundquivst and Figdor’s criteria as it lacks the adhesive quality needed to seal the root canal microscopically. It undergoes shrinkage as it cools after heating for adaptation to obturate the root canal, due to phase transitions. However, it satisfies the remaining requirements and is the preferred filling material for most dentists today [90,91,92]. Being a low-cost, well-studied material, it is no surprise that dentists tend to use gutta-percha more often. This review examined in vitro studies using gutta-percha. It is difficult to extrapolate the results to a clinical setting. However, based on the findings, we can suggest that the commonly used technique of lateral condensation, to obturate root canals with GP may not be ideal and thermoplastic techniques may produce better results. By introducing the thermoplastic techniques as part of the curriculum in more dental schools at the undergraduate level, dentists can be made more adept at using the material better. This could lead to lower failure rates and better, more complete obturations.

## 5. Conclusions

Neither technique of obturation—cold lateral condensation or warm gutta-percha—produced a void-free complete root canal obturation when examined using micro-CT. The thermoplasticized techniques, however, did have significantly fewer voids in most studies. Most studies had a moderate risk of bias and thus the interpretation should be considered with caution. Future studies need to keep in mind the operator skills when comparing techniques such as obturation and introduce blinding in evaluation to achieve less biased results. The introduction of the thermoplastic technique into the dental school curriculum as a pre-clinical exercise may help promote the use of this technique and allow better comparisons in studies.

## Figures and Tables

**Table 1:** Search queries used in the databases.

Database	Query
PubMed	(quality) AND (((root canal) AND ((obturation) OR (filling))) AND (technique)) AND (micro-CT) Details: (“qualities”[All Fields] OR “quality”[All Fields] OR “quality s”[All Fields]) AND ((“dental pulp cavity”[MeSH Terms] OR (“dental”[All Fields] AND “pulp”[All Fields] AND “cavity”[All Fields]) OR “dental pulp cavity”[All Fields] OR (“root”[All Fields] AND “canal”[All Fields]) OR “root canal”[All Fields]) AND (“obturate”[All Fields] OR “obturated”[All Fields] OR “obturating”[All Fields] OR “obturation”[All Fields] OR “obturations”[All Fields] OR “obturator”[All Fields] OR “obturators”[All Fields] OR “obturing”[All Fields] OR (“filled”[All Fields] OR “filling”[All Fields] OR “fillings”[All Fields] OR “fills”[All Fields])) AND (“methods”[MeSH Subheading] OR “methods”[All Fields] OR “techniques”[All Fields] OR “methods”[MeSH Terms] OR “technique”[All Fields] OR “technique s”[All Fields])) AND (“X-ray microtomography”[MeSH Terms] OR (“X-ray”[All Fields] AND “microtomography”[All Fields]) OR “X-ray microtomography”[All Fields] OR (“micro”[All Fields] AND “ct”[All Fields]) OR “micro-CT”[All Fields])
Scopus	TITLE-ABS -KEY ((quality) AND (((root AND canal) AND ((obturation) OR (filling))) AND (technique)) AND (micro AND ct))
Embase	quality AND root AND canal AND (obturation OR filling) AND technique AND micro AND ct
Web of Science	ALL FIELDS: ((quality) AND (((root canal) AND ((obturation) OR (filling))) AND (technique)) AND (micro-CT))

**Table 2:** Data extracted from selected studies.

S. No	Author, Year, (Country)	Samples Used (Size per Group)	Thermoplastic Technique Used	Sealer	Final Irrigants	Measurement Method	Values Obtained		Results
							Cold Lateral Condensation (CLC)	Thermoplastic Technique	
1	Simsek et al., 2017 (Turkey)	Mesial root canals of mandibular first molars (10)	Thermoplasticized injectable gutta-percha (TIGP)	AH plus	17% EDTA, 2.5% NaOCl	Volume of voids and filling (in mm^3^) calculated for both techniques using micro-CT for canals instrumented using self-adjusting files (SAF) and rotary files.	3.11 ± 2.06 mm^3^ (SAF) 2.78 ± 1.20 mm^3^ (Rotary)	3.81 ± 1.87 mm^3^ (SAF) 3.43 ± 0.90 mm^3^ (Rotary)	No significant differences were observed between techniques of preparation and obturation. In the filling techniques, independent of the instrumentation, more voids were in the thermoplastic GP technique, but the differences were not statistically significant.
2	Oh et al., 2016 (Korea)	Permanent mandibular first molars (20)	Continuous wave of condensation (CWC)	AH plus	17 % EDTA, 3.5% NaOCl	The interface void volume ratio of the main canal was also calculated as a percentage of the main canal volume for the apical 5 mm.	0.77 ± 0.16%	0.27 ± 0.12% *	Filling density and adaptation was inferior in CLC filled canals with significantly higher interface void volume ratios. The gutta-percha volume ratio was also significantly lower in CLC than in CWC, but the sealer volume ratio was significantly higher in CLC than in CWC.
3	Ho et al., 2016 (China)	Permanent mandibular first molars (11)	Warm Vertical Compaction (WVC) with Thermoplasticized injectable gutta-percha (TIGP)	None	3% NaOCl, 17% EDTA	The overall mean fraction of the root canal volume filled with gutta-percha for each group was determined	68.51 ± 6.75% filled	88.91 ± 5.16% filled *	The overall gutta-percha volume was significantly lower in the CL group than in the other groups. Within the CL group, the volume fraction was the same in all segments while in WVC, it increased towards the coronal aspect
4	Celikten et al., 2015 (Turkey)	First mandibular premolars (10)	Core carrier technique (Thermafil)	Endo-Sequence BC	2.5% NaOCl, 17% EDTA, distilled water	Using 2D slices, the root filling volume percentages, the volume of internal, external and combined voids in materials was calculated.	0.5 ± 0.2 (internal) 0.8 0.5 (external) 0.6 ± 0.3 (combined)	0.4 ± 0.2 (internal) 0.7 ± 0.4 (external) 0.6 ± 0.3 (combined)	Thermafil had the smallest void volumes (both types) but significantly differed at the apical level only when compared to CLC. The overall volumes were not significantly different for both techniques.
5	Kiekerlo, 2015, Poland	Mandibular premolars (10)	Continuous Wave of Condensation	ZOE-based sealer (Tubli-seal)	17% EDTA, 2% NaOCl and saline solution	The number, size, percentage of volume and distribution of voids-internal (I) and external (E) was measured for groups instrumented with hand (H) and rotary (R) instruments	0.21 ± 0.18% (HI) 0.69 ± 0.41% (HE) 0.27 ± 0.28% (RI) 0.52 ± 0.38% (RE)	0.11 ± 0.12% (HI) ** 0.14 ± 0.13% (HE) 0.20 ± 0.30% (RI) * 0.55 ± 0.48% (RE)	CLC produced voids mainly between the canal wall and the filling. With thermal compaction, the internal voids were more common, except for the apical third of the canal where mostly parietal voids were present.
6	Nhata et al., 2014 (Brazil)	Mandibular incisors (10)	Continuous wave of condensation	AH plus	17% EDTA, Distilled water	The presence of voids at the interface between the root canal dentin and the filling material in all filling techniques investigated	0.019 ± 0.005%	0.004 ± 0.003% *	Less empty spaces were observed when GP was heated within the root canal on continuous wave of condensation compared to CLC.
7	Keleş et al., 2014 (Turkey)	Single rooted maxillary premolars (12)	Thermoplasticized injectable gutta-percha (TIGP)	AH plus	Saline solution	The volume of gutta-percha, sealer and voids was expressed as the percentage of the root canal volume	4.26 ± 0.74%	0.57 ± 0.44% *	The WVC group had a significantly lower percentage volume range of voids (*p* < 0.05) overall but the difference was insignificant for the techniques in the apical thirds
8	Naseri et al., 2013 (Iran)	Maxillary first molars (5)	Warm vertical condensation, Thermoplasticized injectable gutta-percha (TIGP)	AH26 sealer	17% EDTA, 2.5% NaOCl	Expressed as the percentage of the root canal volume	80.4 ± 1.6%	84.8 ± 6.0%—WVC 92.7 ± 2.4% *—TIGP	The TIGP group had the least volume of voids, which was significantly less than both CLC and WVC. Both thermal techniques showed better fill volumes than CLC, which also had a higher percentage of sealer than any other technique.
9	Moeller et al., 2013 (Denmark)	Mandibular molars, premolars, and canines (34—CL and 33 in TH)	Hybrid Thermafil Technique (HT)	AH plus	17% EDTA, 0.5% NaOCl	2D slices were compared for the presence of voids in root fillings by assessing each section (672 µm apart) using a binary value—void present/void not present	65.9% of the sections had voids	66.9% of the sections had voids	A high frequency of voids was found for both techniques, increasing coronally. CLC resulted in fewer voids in the apical part of the root filling. The opposite was true for HT which had more voids cervically. Overall, the two techniques did not differ significantly in the percentage of voids.

* significant difference between groups, ** significant difference between all groups, EDTA—Ethyline Diamine Tetra-acetic Acid, NaOCL—Sodium hypochlorite.

**Table 3:** Assessment of risk of bias.

S No.	Author, Year (Country)	Were Human Teeth Used as Specimens?	Was the Morphology Same for All Root Canals and without Any Root Perforation or Fracture?	Were the Groups Matched?	Was the Rationale for the Sample Size Mentioned?	Was a Standardized Root Canal Preparation and Disinfection Protocol Followed for Both Groups?	Did a Single Operator Perform All Procedures?	Was the Operator Skilled in Both Techniques?	Was the Observer/Evaluator Blind to the Groups?	Was the Entire Root Canal Volume Considered?	Risk of Bias
1	Simsek et al., 2017 (Turkey)	Yes	Yes	Yes	Not mentioned	Yes	Yes	Not mentioned	Not mentioned	Yes	Moderate
2	Oh et al., 2016 (Korea)	Yes	Yes	Yes	Not mentioned	Yes	Yes	Not mentioned	Not mentioned	No	High
3	Ho et al., 2016 (China)	Yes	Yes	Yes	Not mentioned	Yes	Not mentioned	Not mentioned	Not mentioned	Yes	Moderate
4	Celikten et al., 2015 (Turkey)	Yes	Yes	Not mentioned	Not mentioned	Yes	Yes	Not mentioned	Yes	Yes	Moderate
5	Kiekerlo, 2015, Poland	Yes	Yes	Not mentioned	Not mentioned	Yes	Yes	Not mentioned	Not mentioned	Yes	Moderate
6	Nhata et al., 2014 (Brazil)	Yes	Not mentioned	Not mentioned	Not mentioned	Yes	Yes	Not mentioned	Not mentioned	Yes	High
7	Keleş et al., 2014 (Turkey)	Yes	Yes	Yes	Yes	Yes	Yes	Not mentioned	Not mentioned	Yes	Low
8	Naseri et al., 2013 (Iran)	Yes	Yes	Yes	Not mentioned	Yes	Yes	Not mentioned	Not mentioned	Yes	Moderate
9	Moeller et al., 2013 (Denmark)	Yes	Yes	Yes	Not mentioned	Yes	2 operators alternated between techniques	Yes	Yes	No	Moderate

Studies that reported 7–9 of the items were classified as low-risk studies, those which report 5–6 items were considered as moderate bias studies and those with less than 5 reported were considered as having a high risk of bias.
